# Physical Comorbidities in Depression Co-Occurring with Anxiety: A Cross Sectional Study in the Czech Primary Care System

**DOI:** 10.3390/ijerph121215015

**Published:** 2015-12-10

**Authors:** Petr Winkler, Jiří Horáček, Aneta Weissová, Martin Šustr, Martin Brunovský

**Affiliations:** 1 National Institute of Mental Health, Topolová 748, 250 67 Klecany, Czech Republic; jiri.horacek@nudz.cz (J.H.); aneta.weissova@nudz.cz (A.W.); martin.brunovsky@nudz.cz (M.B.); 2 Institute of Psychiatry, Psychology and Neuroscience, King’s College London, 16 De Crespigny Park, London SE5 8AF, UK; 3 Third Medical Faculty, Charles University, Ruská 2411/87, 100 00 Prague, Czech Republic; 4 KRKA Čr, S.r.o, Mezibranská 579/7, 110 00 Prague, Czech Republic; martin.sustr@kzka.biz

**Keywords:** depression, anxiety, comorbidity, primary care, cross-sectional study, prevalence, Czech Republic

## Abstract

Comorbidities associated with depression have been researched in a number of contexts. However, the epidemiological situation in clinical practice is understudied, especially in the post-Communist Central and Eastern Europe region. The aim of this study was to assess physical comorbidities in depression, and to identify whether there are increased odds of physical comorbidities associated with co-occurring depressive and anxiety disorders. Data on 4264 patients aged 18–98 were collected among medical doctors in the Czech Republic between 2010 and 2011. Descriptive statistics were calculated and multiple logistic regressions were performed to assess comorbidities among patients with depressive disorder. There were 51.29% of those who have a physical comorbidity, and 45.5% of those who have a comorbid anxiety disorders among patients treated with depression in Czech primary care. Results of logistic regressions show that odds of having pain, hypertension or diabetes mellitus are particularly elevated at those who have co-occurring depressive and anxiety disorder. Our findings demonstrate that comorbidities associated with depressive disorders are highly prevalent in primary health care practice, and that physical comorbidities are particularly frequent among those with co-occurring depressive and anxiety disorders.

## 1. Introduction

High comorbidity between depressive disorders and anxiety disorders, high comorbidity between anxiety disorders and physical health problems, and high comorbidity between depressive disorders and physical health problems have all been demonstrated in numerous studies [1,2,3,4,5,6]. However, depressive and anxiety disorders are more chronic when occurring together [7,8], and their co-occurrence further elevates the risk of physical problems, such as cardiovascular disorders and hypertension [9,10,11,12], diabetes [12,13], and pain [10,14].

This has important implications for the treatment practice. Petersen *et al.* [15] suggested that more accurate guidelines are needed for those with comorbid depressive and anxiety disorders. This suggestion was made as the investigation into treatment pathways in a Rhode Island (USA) hospital revealed that the only difference between the treatment of those with depressive disorder who developed anxiety, and the treatment of those with depressive disorder who did not develop anxiety disorder, was significantly higher prescription of psychotropics [15].

Epidemiological studies focused on comorbidities associated with depression and anxiety mostly relied on standardized instruments rather than on clinical judgements. It remains a question to what extend do the results of these studies reflect the situation in clinical practice. Also, there is a lack of studies conducted in the post-Communist Central and Eastern Europe (CEE) region [16] and the situation in the primary practice of medical doctors in this area is largely understudied.

For that reason, we took advantage of data collected within a commercial survey pursued by the KRKA Company (Slovenia, Czech Republic) in order to identify treatment habits among the Czech medical doctors in primary care. We analyzed these data with the aim to assess the prevalence of physical comorbidities associated with both, depressive disorders as well as co-occurring depressive and anxiety disorders, among those treated in the Czech primary care system. We particularly aimed to identify whether the comorbid depressive and anxiety disorders elevate odds of having concurrent physical disorders.

## 2. Experimental Section

Data for this multi-center cross-sectional study were collected from medical doctors in primary care in the Czech Republic. According to the Czech Medical Chamber, there are 1497 neurologists, 1347 psychiatrists and 6794 general practitioners in the Czech Republic. Convenient sampling method was used to approach these medical doctors and to achieve reasonably representative sample in terms of their regional distribution and usual treatment settings. Medical doctors were approached by representatives of the KRKA Pharmaceutical Company and offered a possibility to participate in the research on their therapeutic habits with respect to patients with depression. Although the precise number is not available, based on the representatives’ statements the response rate was reported to be about 80%. This included general practitioners (n = 34), psychiatrists (n = 63), and neurologists (n = 33) who all are allowed to prescribe antidepressants in the Czech Republic. These medical doctors were asked to use their own registers and to report on patients who are currently treated with depression in their practice. 

The questionnaire was focused on comorbidities among patients with mild (F32.0 or F33.0), moderate (F32.1 or F33.1), and severe (F32.2 or F33.2) depressive disorder, as well as on patient with severe depressive disorder with psychotic symptoms (F32.3 or F33.3) as classified by ICD-10. The questionnaire was distributed in electronic form. Included were questions on selected comorbid anxiety disorders, and questions on selected comorbid physical health problems. Anxiety disorders included generalized anxiety disorders (F41.1), panic disorders (F41.0), post-traumatic stress disorders (F43.1), social phobia (F40.1), agoraphobia (F40.0) and other phobias (F40.2 or F40.8), but not other types of anxiety disorders. Physical health problems included pain, hypertension, diabetes mellitus, cardiovascular diseases other than hypertension, neoplasms, organic diseases of nervous system, endocrine diseases, and gastrointestinal diseases, but not other types of physical health problems. All of the questions on comorbidities had a dichotomous character, and medical doctor was asked to state whether a patient was or was not treated for the particular condition. The presence of both, anxiety disorders as well as physical conditions, were reported by medical doctor according to his or her medical records. This means that data refer to actual clinical practice and reflect those conditions which a patient has been treated for. To understand how medical conditions were diagnosed in this study, a contextual reference to Czech health care is needed. All diagnoses within the Czech health care system are based on the 10th revision of the International Statistical Classification of Diseases and Related Health Problems (ICD-10). Czech law obliges all medical doctors to obtain education which enables them to use ICD-10 codes. Also, diagnosis which is not based on ICD-10 operational criteria and does not have a code described in this classification system is neither accepted by the health care authorities, nor reimbursed by health insurance companies. This means that all diagnoses reported in this paper are backed by ICD-10; however, we did not refer to a specific ICD-10 code when we used a broader category, such as pain, which includes a number of different ICD-10 codes.

The Mantel-Haenszel method was used to stratify the odds of having each of the physical comorbidities by age and to test for possible interactions. Associations between all physical comorbidities and patients’ sociodemographic characteristics were tested using chi-square tests. Sociodemographic characteristics that were identified as significantly influencing the distribution of comorbidities were included into regression models. Multiple logistic regression models were built in order to examine odds of having physical comorbidity while having co-occurring depressive and anxiety disorders as opposed to having depressive disorder only. Logistic regressions model was built for each of the physical conditions separately while controlling for gender, age, number of depressive episodes during the life time, type of depressive episode (e.g., F32.0, F32.1, F32.2, or F32.3), and length of the current depressive episode. Statistical analyses were performed in Stata 11 (StataCorp LP, College Station, TX, USA).

## 3. Results and Discussion

### 3.1. Participants

Data on 4264 patients with depression were gathered during January 2010 and December 2011. Since only adults were eligible for the study, the minimum age in the study sample was 18, mean age was 44.8 and maximum age was 98 years. There were 58% of females and 42% of males in our sample. 

The respondents differed according to whether their depressive episode was mild (F32.0 or F33.0; n = 1929; 45.24%), moderate (F32.1 or F33.1; n = 1940; 45.50%), severe (F32.2 or F33.2; n = 354; 8.30%) or severe with psychotic symptoms (F32.3 or F33.3; n = 41; 0.96%); according to whether it was their first (n = 2543; 59.65%), second (n = 1255; 29.4%) third (n =285; 6.7%), or fourth or further (n = 181; 4.25%) episode of depression; and according to whether the current depressive episode lasted for less than a month (n = 757; 17.75%), one to three months (n = 2021; 47.40%), three to six months (1087; 25.49%), six to twelve months (n = 282; 6.61%), or longer (n = 117; 2.74%, Table 1). Most of the patients were treated by psychiatrists (Table 2).

**Table 1:** Participants’ characteristics stratified by the type of depressive episode.

Type of the Depressive Episode	Mild	Moderate	Severe	Severe with Psychotic Symptoms	Total
Gender					
Men	824	808	151	19	1802
Women	1105	1132	203	22	2462
Age					
18–34 years	644	469	61	7	1181
35–49 years	824	665	113	13	1615
50–65 years	304	513	95	9	921
>65 years	157	293	85	12	547
Lifetime number of depressive episodes					
1	1589	869	78	7	2543
2	278	832	134	11	1255
3	33	149	90	13	285
>4	29	90	52	10	181
Length of the current depressive episode					
<1 month	550	168	35	4	757
1–3 months	918	972	116	15	2021
3–6 months	325	615	139	8	1087
6–12 months	90	132	50	10	282
>12 months	46	53	14	4	117

**Table 2:** Description of the sample with respect to the treatment setting.

Number of People with Depression	Neurologists	Psychiatrists	GPs	Total
<30	12	29	30	71
30–74	11	5	16	32
75–149	9	17	10	36
150–299	3	20	5	28
>300	5	28	1	34
Total	40	99	62	201

### 3.2. Outcomes

Out of all respondents, 788 (18.52%) had comorbidity with generalized anxiety disorder, 555 (13.04%) with panic anxiety disorder, 293 (6.89%) with post-traumatic stress disorder, 405 (9.52%) with social anxiety disorder, 137 (3.22%) with agoraphobia, and 90 (2.12%) with some other type of phobia. 241 respondents had a comorbidity with more than one type of anxiety disorder, and there were 2,324 (54.50%) of respondent with depression only. 

Regarding to physical comorbidities, 2077 (48.71%) respondents had no physical comorbidity; 985 (23.10%) had a comorbidity with pain; 904 (21.20%) with hypertension; 373 (8.75%) with diabetes; 293 (6.87%)with cardiovascular problems other than hypertension; 147 (3.45%) with neoplasms; 184 (4.32%) with organic diseases of central nervous system, 151 (3.54%) with endocrine diseases, and 254 (5.96%) with gastrointestinal diseases. 1462 (34.29%) of patients had just one physical comorbidity, 467 (10.95%) had two physical comorbidities, 172 (4.03%) had three physical comorbidities, and 86 (2.02%) had four or more physical comorbidities. Prevalence of physical comorbidities among those with concurrent depressive and anxiety disorder is given in the Table 3.

In our sample, when compared to depression without co-occurring anxiety disorders, depression co-occurring together with GAD was found to be associated with elevated odds of pain and gastrointestinal diseases; depression co-occurring together with panic disorder was found to be associated with elevated odds of pain and neoplasms; depression co-occurring together with PTSD was found to be associated with elevated odds of pain, hypertension, and diabetes mellitus; depression co-occurring together with social anxiety disorder was found to be associated with elevated odds of diabetes, cardiovascular diseases other than hypertension, and gastrointestinal diseases; depression co-occurring together with agoraphobia was found to be associated with elevated odds of hypertension, and diabetes; and depression co-occurring together with other phobias was found to be associated with elevated odds of hypertension and diabetes. While Table 4 shows crude odds ratios for physical comorbidities among those with co-occurring depressive and anxiety disorders, Table 5 demonstrates results of logistic regression where we controlled for age, gender, type of depressive episode, number of depressive periods during the lifetime, and the length of the current depressive disorder.

**Table 3:** Prevalence of physical comorbidities among those with depressive disorder co-occurring with anxiety disorder.

Prevalence of Comorbidity	GAD		Panic Dis.		PTSD		SAD		Agora Phob.		Other Phob.	
	Yes	No	Yes	No	Yes	No	Yes	No	Yes	No	Yes	No
Pain	315 (40%)	474 (60%)	182 (33%)	373 (67%)	108 (37%)	185 (63%)	86 (21%)	320 (79%)	25 (18%)	112 (82%)	14 (16%)	76 (84%)
Hypertension	207 (26%)	582 (74%)	110 (20%)	445 (80%)	81 (28%)	212 (72%)	93 (23%)	313 (77%)	36 (26%)	101 (74%)	26 (29%)	64 (71%)
Diabetes Mellitus	77 (10%)	712 (90%)	49 (9%)	506 (91%)	39 (13%)	254 (87%)	62 (15%)	344 (85%)	24 (18%)	113 (82%)	13 (14%)	77 (86%)
Cardiovascular	66 (8%)	723 (92%)	42 (8%)	513 (92%)	27 (9%)	266 (91%)	48 (12%)	358 (88%)	14 (10%)	123 (90%)	7 (8%)	83 (92%)
Neoplasms	39 (5%)	750 (95%)	32 (6%)	523 (94%)	18 (6%)	275 (94%)	17 (4%)	389 (96%)	5 (4%)	132 (96%)	3 (3%)	87 (97%)
Organic Diseases of Nervous System	44 (6%)	745 (94%)	18 (3%)	537 (97%)	12 (4%)	281 (96%)	26 (6%)	380 (94%)	6 (4%)	131 (96%)	3 (3%)	87 (97%)
Endocrine Diseases	35 (4%)	754 (96%)	19 (3%)	536 (97%)	11 (4%)	282 (96%)	25 (6%)	381 (94%)	10 (7%)	127 (93%)	3 (3%)	87 (97%)
Gastrointestinal Diseases	70 (9%)	719 (91%)	43 (8%)	512 (92%)	25 (9%)	268 (91%)	41 (10%)	365 (90%)	8 (6%)	129 (94%)	6 (7%)	84 (93%)
Total n=4.264 (100%)	789 (18.5%)		555 (13.0%)		293 (6.9%)		406 (9.5%)		137 (3.2%)		90 (2.1%)	

**Table 4:** Depression with co-occurring anxiety disorders, crude odds ratios for physical comorbidities.

**Comorbidity**	**Pain**			**Hypertension**			**Diabetes Mellitus**			**Cardiovascular**		
	**OR**	***p***	**95% CI**	**OR**	***p***	**95% CI**	**OR**	***p***	**95% CI**	**OR**	***p***	**95% CI**
GAD	2.78	<0.001	2.35–3.29	1.42	<0.001	1.18–1.70	1.16	0.265	0.89–1.51	1.31	0.066	0.98–1.74
Panic Disorder	1.77	<0.001	1.45–2.14	0.91	0.394	0.73–1.13	1.01	0.942	0.74–1.39	1.13	0.487	0.80–1.58
PTSD	2.06	<0.001	1.60–2.64	1.46	0.005	1.12–1.91	1.67	0.004	1.17–2.39	1.41	0.100	0.93–2.14
SAD	0.88	0.335	0.69–1.14	1.12	0.377	0.87–1.43	2.06	<0.001	1.53–2.76	1.98	<0.001	1.42–2.75
Agora Phobia	0.74	0.171	0.47–1.14	1.34	0.140	0.91–1.97	2.30	<0.001	1.46–3.62	1.57	0.115	0.89–2.77
Other Phobia	0.61	0.086	0.34–1.08	1.53	0.071	0.96–2.42	1.79	0.053	0.98–3.25	1.15	0.731	0.53–2.50
**Comorbidity**	**Neoplasms**			**Organic Diseases of Nervous System**			**Endocrine Diseases**			**Gastrointestinal Diseases**		
	**OR**	***p***	**95% CI**	**OR**	***p***	**95% CI**	**OR**	***p***	**95% CI**	**OR**	***p***	**95% CI**
GAD	1.62	0.011	1.11–2.36	1.41	0.053	0.99–1.99	1.34	0.132	0.91–1.98	1.74	<0.001	1.31–2.32
Panic Disorder	1.91	0.001	1.28–2.86	0.72	0.183	0.44–1.17	0.96	0.872	0.59–1.57	1.39	0.056	0.99–1.96
PTSD	1.95	0.009	1.17–3.24	0.94	0.848	0.52–1.71	1.07	0.838	0.57–2.00	1.52	0.054	0.99–2.35
SAD	1.25	0.391	0.75–2.10	1.60	0.030	1.04–2.46	1.94	0.003	1.25–3.02	1.92	<0.001	1.35–2.73
Agora Phobia	1.06	0.895	0.43–2.64	1.02	0.970	0.44–2.34	2.23	0.016	1.14–4.33	0.98	0.953	0.47–2.02
Other Phobia	0.97	0.952	0.30–3.09	0.76	0.643	0.24–2.43	0.94	0.914	0.29–3.00	1.13	0.774	0.49–2.61

**Table 5:** Depression with co-occurring anxiety disorders; logistic regression of physical comorbidities, controlled for age, gender, type of depressive episode, number of depressive periods during the lifetime, and the length of the current depressive disorder.

**Comorbidity**	**Pain**			**Hypertension**			**Diabetes Mellitus**			**Cardiovascular**		
	**OR**	***p***	**95% CI**	**OR**	***p***	**95% CI**	**OR**	***p***	**95% CI**	**OR**	***p***	**95% CI**
GAD	2.49	<0.001	2.10-2.96	1.19	0.095	0.97–1.45	0.86	0.308	0.65–1.15	1.06	0.731	0.77–1.44
Panic Disorder	1.75	<0.001	1.43-2.15	0.94	0.662	0.73–1.22	0.9	0.561	0.63–1.28	1.15	0.468	0.78–1.68
PTSD	1.87	<0.001	1.45-2.43	1.55	0.004	1.15–2.10	1.63	0.013	1.11–2.39	1.49	0.081	0.95–2.34
SAD	0.76	0.042	0.59-0.99	0.91	0.501	0.67–1.20	1.71	0.001	1.23–2.37	1.64	0.008	1.14–2.36
Agora Phobia	0.71	0.136	0.45-1.11	1.61	0.034	1.04–2.50	2.51	<0.001	1.51–4.18	1.75	0.079	0.94–3.27
Other Phobia	0.6	0.086	0.33-1.07	1.75	0.034	1.04–2.92	2.22	0.013	1.18–4.20	1.35	0.469	0.60–3.05
**Comorbidity**	**Neoplasms**			**Organic Diseases of Nervous System**			**Endocrine Diseases**			**Gastrointestinal Diseases**		
	**OR**	***p***	**95% CI**	**OR**	***p***	**95% CI**	**OR**	***p***	**95% CI**	**OR**	***p***	**95% CI**
GAD	1.25	0.263	0.85–1.84	1.11	0.572	0.77–1.60	1.05	0.794	0.71–1.57	1.5	0.006	1.12–2.02
Panic Disorder	1.63	0.025	1.06–2.50	0.64	0.102	0.38–1.09	0.78	0.337	0.48–1.29	1.26	0.198	0.89–1.78
PTSD	1.66	0.058	0.98–2.82	0.89	0.725	0.48–1.67	0.87	0.675	0.46–1.65	1.39	0.146	0.89–2.15
SAD	0.98	0.941	0.57–1.68	1.31	0.247	0.83–2.06	1.55	0.059	0.98–2.44	1.59	0.012	1.11–2.28
Agora Phobia	0.93	0.886	0.37–2.38	1.2	0.799	0.47–2.67	1.7	0.134	0.85–3.39	0.76	0.46	0.36–1.59
Other Phobia	1.14	0.823	0.35–3.71	0.82	0.739	0.25–2.67	0.98	0.975	0.30–3.17	1.21	0.664	0.52–2.81

### 3.3. Discussion

The findings of our study demonstrate a high prevalence of both physical and anxiety-related psychiatric comorbidities among primary care patients with depression. Our results are of particular interest for primary care settings because annual occurrence of depression has been reported among 6%–10.3% of primary care patients [17,18,19,20]. In addition, a fourth of primary health care utilizers have been found to suffer from current major depression and a fifth from some of the anxiety disorders [21]. In our sample, about a half of subjects suffered from the comorbid depressive and anxiety disorders. Furthermore, about a half of depressive patients had some physical comorbidity, with pain being the most frequent complaint in this regard. We have also found that anxiety disorders co-occurring with depression are associated with increased odds of somatic comorbidities. Large sample size enabled us to have a detailed look into what types of anxiety disorders occurring together with depressive disorders elevate the risks of having pain, hypertension and other cardiovascular problems, diabetes and other physical problems.

The high co-occurrence of depression and anxiety disorders identified in our sample corresponds to the previously published large surveys conducted in the primary care setting. These were reviewed by Hirschfeld [22] who demonstrated that depression is rarely occurring alone, rather it is frequently complicated by other psychiatric comorbidity. Among patients who meet criteria for major depression, approximately 50% in the community [23] and 75% in a primary care setting [24] were found to be suffering from a concurrent anxiety disorder. Those with major depression are 3.3–8.2 times more likely to have a comorbid anxiety disorder [23]. Congruently with our findings, the GAD (followed by PTSD, panic disorder, social phobia and agoraphobia) is the most frequent comorbidity in MDD [23]. 

Previous cross-sectional studies have consistently reported associations between major depression and chronic physical conditions [25,26,27,28]. However, the design of cross-sectional studies, including the present one, does not allow establishing the direction of the causal link between comorbid physical disorder and mental conditions. While most qualitative studies find that patients interpret their chronic physical condition as contributing to depression or anxiety, some studies find patients expressed the belief that anxiety or depression led to their chronic disease [27]. The former approach to the comorbidity is supported by the findings that the comorbid depression is twice higher in patients with any preceding chronic medical condition (9.2%) than in control group (4.0%) [19]. The latter possibility has received relatively little attention in the literature. However, the data from longitudinal Canadian cohort showed that major depression at the baseline elevated risk for several long-term medical conditions including pain-related problems (1.9-fold for arthritis, 1.4-fold for back pain and 1.9-fold for migraines). This finding corresponds with our result of pain as the most frequently reported medical complain of patients with depression. Also other medical comorbidities (hypertension and other cardiovascular problems, endocrine diseases including diabetes and gastrointestinal diseases) identified in our sample are congruent with findings of this longitudinal cohort [17]. 

Our findings complement previously identified spectrum of clinical consequences of depression comorbid with anxiety disorders comprising high chronicity and severity of both psychiatric conditions, greater impairment in work and psychosocial functioning, lower quality of life, and increased rate of psychiatric hospitalization and suicide attempts [22]. In addition, the results of logistic regressions also point out to specific patterns of physical comorbidities in different anxiety disorders co-occurring with depression. It is not surprising that pain was increased in comorbid GAD, panic disorder and PTSD because pain is frequent in both depression [17,23] and anxiety disorders [29,30]. The population surveys in 17 countries confirmed that GAD, panic disorder and/or agoraphobia, SAD and PTSD were associated with multiple or single site pain problems [30]. Moreover, the same sample also showed, congruently with our findings, that depression comorbid with anxiety disorders is more strongly associated with chronic pain conditions (but not all physical conditions) than non-comorbid depressive or anxiety disorder alone [10].

Interestingly, PTSD, SAD, agoraphobia and other phobias have shared the physical comorbidities related directly (hypertension and other cardiovascular disorders) or indirectly (diabetes mellitus) to the increased sympathetic activity. This finding is in line with the recently formulated integrative hypothesis of common pathophysiological mechanism for affective, cardiovascular and metabolic disorders [31]. The fact that gastrointestinal disorders were associated with GAD and SAD in patients with depression indicates that freely floating anxiety (typical for both GAD and SAD) could predispose for gastrointestinal comorbidities in depression. This assumption should be tested in future longitudinal studies. However, it has been documented by a meta-analytical approach that medically unexplained gastrointestinal problems like irritable bowel or non-ulcer dyspepsia represent the most frequent complications of both depression and anxiety [28]. 

The cross-sectional design of our study does not allow assessing the causal link between depression and physical disorders. Considering the direction of causality, the chronic somatic problems associated with functional limitation, disability or pain may lead to the emergence of an emotional distress initiating the depressive episode [32]. Depression (and anxiety) may also result from the stress and fear produced by medical consultations and investigations (iatrogenic effect) for chronic physical disorder or from the pharmacotherapy used in its treatment [33]. The reverse causal direction is also likely. Depression lowers the threshold for pain, activates stress hormonal response [33], worsens cardiovascular parameters including hypertension [34], and increases the risk for type II diabetes [26]. Interestingly, the comorbid depressive-anxiety disorders are more strongly associated with physical comorbidities than non-comorbid depressive or anxiety disorder alone [10]. This finding, which is fully congruent with our results, supports a causal direction from affective to physical disorders. Therefore, the bidirectional relationship between depression and chronic medical conditions is the most likely mechanism. This explanation would also fit into observation presented in this paper. However, there is also a possibility that depression and physical disorders might have a shared origin to some extent. This hypothesis is supported by our previous studies which demonstrated that the activity of serotonin and other monoamines in brain are directly influencing insulin sensitivity in the periphery [35,36]. This association between central monoaminergic activity and peripheral metabolic parameters could mediate the clinically highly relevant association between type II diabetes mellitus and depression [26]. 

The results reported here are limited in four ways. Firstly, the cross-sectional design limits causal interpretations and does not allow us to establish the interactions and sequence of comorbid disorders. Secondly, the method of data collection, imply that there is a risk of selection bias associated with this study. Data from the large sample of primary care medical doctors in the Czech Republic were gathered within the commercial survey of KRKA Pharmaceutical Company and this might have influenced the data collection process. The research team, however, worked completely independently and was driven by nothing but a professional curiosity. Thirdly, the diagnostic process was based solely on clinical judgements of ICD-10-trained Czech primary care medical doctors, which has both, pros and cons. Fourthly, no socioeconomic data were collected within the study, and so could not be controlled for within our analyses. Also, no information was available as with respect to what extent the data reported by medical doctors matched the data within their registers. Some discrepancies might have occurred and we were not able to quantify them. Finally, we were able to provide only approximate numbers as with regard to how many patients were treated by psychiatrists, how many by neurologists, and how many by general practitioners. Together, the greatest advantage is that this survey mirrors the situation in a clinical practice; the greatest disadvantage is that this might have introduced some discrepancies between what is diagnosed by medical doctors and what would be diagnosed via standardized epidemiological instruments. 

## 4. Conclusions

Our findings demonstrate the high degree of physical and anxiety-related psychiatric comorbidities in patients with depression in the Czech primary care. About half of depressive patients suffered from the comorbidity with anxiety disorders, and the same portion of subject reported physical comorbidities with pain as the most frequent complaint. Logistic regressions show that odds of having pain, hypertension or diabetes mellitus are particularly elevated at those who have co-occurring depressive and anxiety disorder. These odds differ according to the type of anxiety disorder present.

The findings of this study provide further insights into the prevalence of physical comorbidities among those with depressive disorders. Effective recognition and treatment of anxiety and depression may be associated with functional improvement in the medical disorders and may also positively impact the economic burden of these disorders. 
